# Classification of Center of Mass Acceleration Patterns in Older People with Knee Osteoarthritis and Fear of Falling

**DOI:** 10.3390/ijerph191912890

**Published:** 2022-10-08

**Authors:** Arturo González-Olguín, Diego Ramos Rodríguez, Francisco Higueras Córdoba, Luis Martínez Rebolledo, Carla Taramasco, Diego Robles Cruz

**Affiliations:** 1 Centro de Estudios del Movimiento Humano (CEMH), Escuela de Kinesiologia, Facultad de Salud y Odontologia, Universidad Diego Portales, Santiago 8370109, Chile; 2 Escuela de Kinesiologia, Facultad de Salud y Ciencias Sociales, Universidad de Las Americas, Santiago 7500975, Chile; 3 Hospital Regional Rancagua, Rancagua 2820000, Chile; 4 ELEAM Ayen Ruca, Cunco 4890000, Chile; 5 Carrera de Kinesiología Universidad Mayor, Santiago 8580745, Chile; 6 Facultad de Ingenieria, Universidad Andres Bello, Vina del Mar 2531015, Chile; 7 Millennium Nucleus on Sociomedicine, Las Condes 7560908, Chile; 8 Escuela de Ingeniería Civil Informática, Universidad de Valparaíso, Valparaíso 2362905, Chile; 9 Carrera de Kinesiología, Facultad de Ciencias de la Salud, Universidad Central de Chile, Santiago 8330546, Chile

**Keywords:** preoccupation, fall, knee osteoarthritis, acceleration, gait, deep learning

## Abstract

(1) Background: The preoccupation related to the fall, also called fear of falling (FOF) by some authors is of interest in the fields of geriatrics and gerontology because it is related to the risk of falling and subsequent morbidity of falling. This study seeks to classify the acceleration patterns of the center of mass during walking in subjects with mild and moderate knee osteoarthritis (KOA) for three levels of FOF (mild, moderate, and high). (2) Method: Center-of-mass acceleration patterns were recorded in all three planes of motion for a 30-meter walk test. A convolutional neural network (CNN) was implemented for the classification of acceleration signals based on the different levels of FOF (mild, moderate, and high) for two KOA conditions (mild and moderate). (3) Results: For the three levels of FOF to fall and regardless of the degree of KOA, a precision of 0.71 was obtained. For the classification considering the three levels of FOF and only for the mild KOA condition, a precision of 0.72 was obtained. For the classification considering the three levels of FOF and only the moderate KOA condition, a precision of 0.81 was obtained, the same as in the previous case, and finally for the classification for two levels of FOF, a high vs. moderate precision of 0.78 was obtained. For high vs. low, a precision of 0.77 was obtained, and for the moderate vs. low, a precision of 0.8 was obtained. Finally, when considering both KOA conditions, a 0.74 rating was obtained. (4) Conclusions: The classification model based on deep learning (CNN) allows for the adequate discrimination of the acceleration patterns of the moderate class above the low or high FOF.

## 1. Introduction

According to data and reports from the World Bank and the WHO, a large part of the population has increased its life expectancy steadily, reaching 75 years or more in many countries [1]. It is estimated that life expectancy has practically tripled in the last century. In this sense, government policies have placed emphasis and attention on measures that can prevent problems arising from aging implementing public policies focused on promotion, prevention, empowerment, and rehabilitation strategies throughout the life course [2,3,4].

During the aging process, impairments or dysfunctions can be identified that affect both cognitive and physical health, among others, including Alzheimer’s disease, depression, neurological sequelae, cardiorespiratory disorders, or degenerative musculoskeletal conditions [5,6,7]. In elderly people (EP), this implies consequences in their relationship with the environment which leads to the appearance of barriers to participation and social interaction as well as deterioration in quality of life. Addressing these functional impairments, activity limitations or participation restrictions requires that they be approached from different analysis perspectives in order to try to anticipate major complications that threaten autonomy and health status [8,9].

The fear of falling (FOF) that arises when moving or after suffering a fall has various conceptual nuances according to the different perspectives of the authors (kinesiophobia, FOF, etc.). In this research paper, we will refer in general terms to FOF [10,11,12]. It can be defined as a cognitive–perceptual state in which there is the idea of a risk of falling (when standing up or walking), generating avoidance behaviors to move, and thus limiting interaction with the environment, restricting movement precisely to avoid falling [13,14,15,16,17]. Although the preoccupation related to the fall is usually something that can actually occur in specific situations (such as unstable surfaces, degree of movement difficulty, and use of cognitive resources, among others), certain conditions have been studied that can exacerbate this condition, such as status cognitive, history of falls, mood, physical performance, and comorbidities [18,19]. It is estimated that about 60% of EP fallers show FOF. Frequently, this may be present with other comorbidities and may worsen the restriction and relationship of EP in the community. This indicator seems to be relevant, because the evidence suggests that falls in PM show a correlation with morbidity and mortality, loss of functionality, and early admission to long-term care homes [20].

A frequent comorbidity linked to the risk of falls presented by EP is knee osteoarthritis (KOA), which may have a multifactorial origin, although its prevalence increases with age, manifesting in a loss of articular cartilage integrity and optimal functionality of the knee with different levels of severity [21,22].

KOA affects most people over the age of 65, being more prevalent in women. Some characteristics of this clinical disease such as pain, decreased strength, vulnerability to balance reactions, limited arthrokinematics and osteokinematics of the knee by stiffness or pain, or structural degeneration, could increase the risk of falling, generating postural transfer patterns or cautious gait patterns [23,24,25]. It is possible that it contributes to worsening the patterns of functionality associated with movement, such as walking.

Considering that in EP the FOF together with the KOA are probably facilitating factors of the risk of falling, the question arises about how the different levels of FOF and OAR affect or harm the magnitude and structure of the movement, manifesting in a lower adaptability and the consequential cautious pattern when moving [26,27,28,29]. Gait is considered an indicator of functionality and by some authors as the sixth vital sign [30,31]. Therefore, seen from the perspective of gait analysis, it is possible to infer that in EP with KOA we will precisely find typical temporal and spatial values (such as gait speed, cadence, step length, and amplitude of movement, among others) to be less than in EP without KOA [32,33,34,35].

An alternative that can strengthen the analysis and interpretation of the results obtained from data obtained from biomechanical variables may be machine learning algorithms, which can delve into possible relationships that arise in and between the data typically obtained from discrete biomechanical variables. In particular, deep learning is an approach that can greatly contribute to the understanding of motor behavior to model human movement in a time series. Additionally, it would be beneficial to gain a more complete understanding of the mechanics of gait and its underlying causes of dysfunction [36].

In particular, convolutional neural networks (CNNs) are algorithms that can be used in multiple fields, and have been extended as a great novelty to the study of human movement [37], being able to extract and analyze data from hierarchically basic and complex sources that occur in a temporal series; that is, they can learn from their non-linear and temporal relationships, training themselves and generating groupings and convolutions in such a way that the convolutional layers extract particular characteristics in different locations of their inputs. Using classification algorithms such as CNNs is interesting, considering the complex nature of human movement, and thus it is an alternative approach to potential non-trivial explanations of human movement [38].

Therefore, the main objective of this research focuses on classifying the acceleration patterns of the center of mass of the march in PD with KOA that present different levels of preoccupation related to the fall.

## 2. Materials and Methods

A total of 78 independent EP were recruited in the community through a sample of volunteer participants and snowballing from different healthy living workshops for EP. Among the selection criteria was the medical diagnosis of KOA mild or moderate (unilateral or bilateral), minimental test with values within typical ranges, as well as being independent according to the Barthel scale. EP with knee prostheses, severe KOA, or who were experiencing some temporary or chronic medical condition that affected walking, as well as sensory or motor conditions that could be a confounding factor, were excluded. The final sample was made up of 69 participants, and details and characteristics can be found in Table 1.

### 2.1. Fear or Preoccupation Related to the Fall

Fear of falling (FOF) or preoccupation related to the fall was assessed by using the Short FES-I [39]. This is an abbreviated version of the International Falls Efficacy Scale to assess fear or preoccupation with falling. The EP must answer some questions about how worried they are about the possibility of falling by thinking about how they usually perform some activities such as getting dressed, taking a bath or shower, getting up from a chair, going up or down stairs, reaching for something overhead, going up or down a slope, and finally going out to a social event. The response options were not at all worried, somewhat worried, quite worried, and very worried [40].

The classification scores for FES-1 used in this research are those applied in [15] 7–8: low FOF; 9–13: moderate FOF; and 14–28: high FOF (see Table 2).

### 2.2. Gait Assessment Procedure

Participants were asked to walk at their self-selected speed along a 30-m flat surface with the instruction “walk straight at your usual, comfortable pace,” similar to that in other studies that have reported the gait study by using accelerometry [41]. The walking tests were recorded by using a triaxial accelerometer from a Samsung Galaxy S8 smartphone [42]. This tool presents high validity (r of 0.89) and reliability (ICC of 0.9) for the study of gait when compared to traditional accelerometry. The mobile device was firmly fixed with a velcro belt at the vertebral level of L5. All tests were performed on a flat, illuminated surface. The first and last five spikes of the acceleration signals (initial and final five meters of the record) were eliminated in order to analyze approximately 20 m located in the center of each walking test in order to avoid acceleration and deceleration accommodations. Accelerometry is one of the most widely used gait measurement tools today. It belongs to the family of inertial devices, which are capable of measuring linear acceleration or angular velocity [43,44]. They can make 3D motion measurements without the need for external references as other measurement systems require, nor do they require special facilities. They are a technological tool of universal access, reduced size, and low cost, and are a reliable method for the study of bipedal balance and gait in general [45], allowing for the adequate evaluation of the changes generated by aging in the gait pattern [46]. It is a reliable method for the study of bipedal balance and gait [47].

### 2.3. CNN Model

The acceleration signals were directly fed into the network; each vector has 240 values from channel 1, channel 2, and channel 3, which differs from the traditional machine learning approach based on the use of previously estimated features as inputs.The total data was separated into training, validation, and test data in percentages of 60, 20, and 20, respectively.

**Figure 1:**
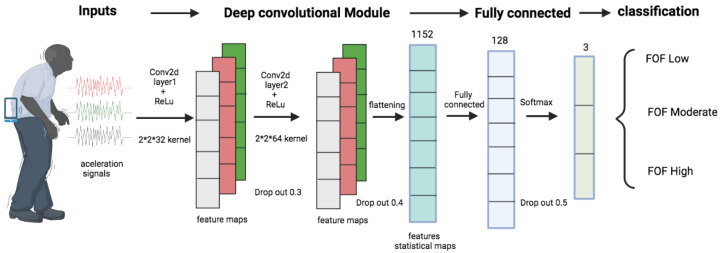
Diagram of the CNN model.

The convolutions layers consist of taking groups of data from the acceleration time series and mathematically operating a scalar product against a small matrix called a kernel (size 2 × 2) which runs through all the input signals (from left to right) and generates a new output matrix, which will ultimately be our new input for the following layers of the network (see Figure 1 and Table 3).

The first convolutional layer contains 32 filters (size 2 × 2 and with a ReLU-type activation function), and the second layer contains 64 filters (size 2 × 2 and ReLU-type activation function), which allows for the extraction of features and patterns each time of more complex acceleration signals.

The ReLU activation function will generate an output equal to zero when the input is negative and an output equal to the input when the latter is positive, thus allowing no saturation.

The flatten function allows flattening, that is, it stops being a three-dimensional volume and becomes a vector that will pass to the traditional hidden layer in which there are 128 neurons, all connected to each other with a ReLU activation function. Subsequently, it passes to a second dense layer which contains three neurons corresponding to the classes that we are classifying with a Softmax type activation function (which returns the probability of each class).

Dropout is a technique by which randomly selected neurons are ignored during training. This means that their contribution to the firing of neurons that follow in the chain is temporarily removed in the forward step, and weight updates are not applied to the neuron in the backward step. This results in a network that is better able to generalize and is less likely to overfit the training data.

The CNN model was implemented in Google Colab by using the Tensorflow Keras library [48] on a standard computer.

## 3. Results

The final sample consisted of 69 participants (mean age 74.18 +/− 5.44) (details in Table 1. All EPs were functionally independent. A total of 46 participants presented mild KOA (low FOF (13), moderate FOF (16), and high FOF (17), and 23 moderate KOA participants (low FOF (4), moderate FOF (6), and high FOF (13). All participants showed homogeneous characteristics in their variables that represent them. In the case of the Barthel score, they presented variability, due to the broad score that characterizes this scale. Despite the above, all participants were independent.

The scores that characterize the different levels of FOF observed in Table 2 show low FOF levels in 17 participants (KOA mild (13) and KOA moderate (4)), a moderate level of FOF (22) (KOA mild (16) and KOA moderate (6)), and a high level of FOF (30) participants (KOA mild (17) and KOA moderate (3)).

**Figure 2:**
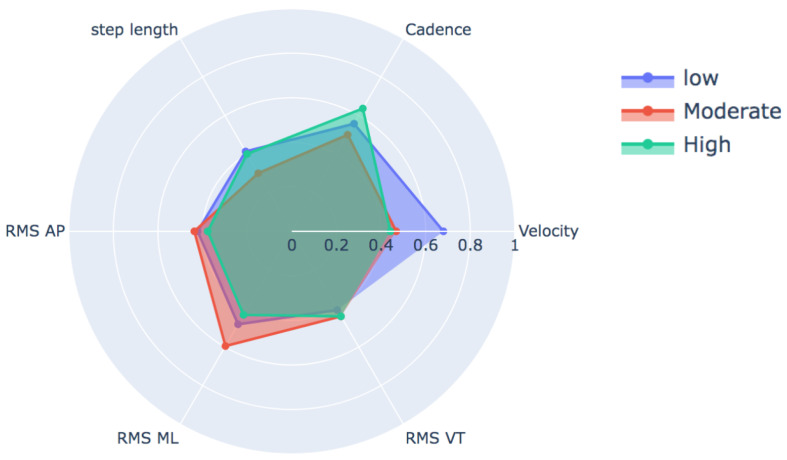
Gait characteristics according to degree of FOF. Variable values expressed in z-score.

Figure 2 and Table 4 shows the behavior of variables typically reported in the description of the gait, such as speed, cadence, step length, and root mean square (RMS) according to the degree of FOF. Even though these variables were not used to train our classification model, this graph allows us to appreciate the minimal difference in the behavior of each variable for the condition of interest in this study (FOF).

The results are expressed through the confusion matrix, precision and loss function obtained from the test group for the following conditions.

**Figure 3:**
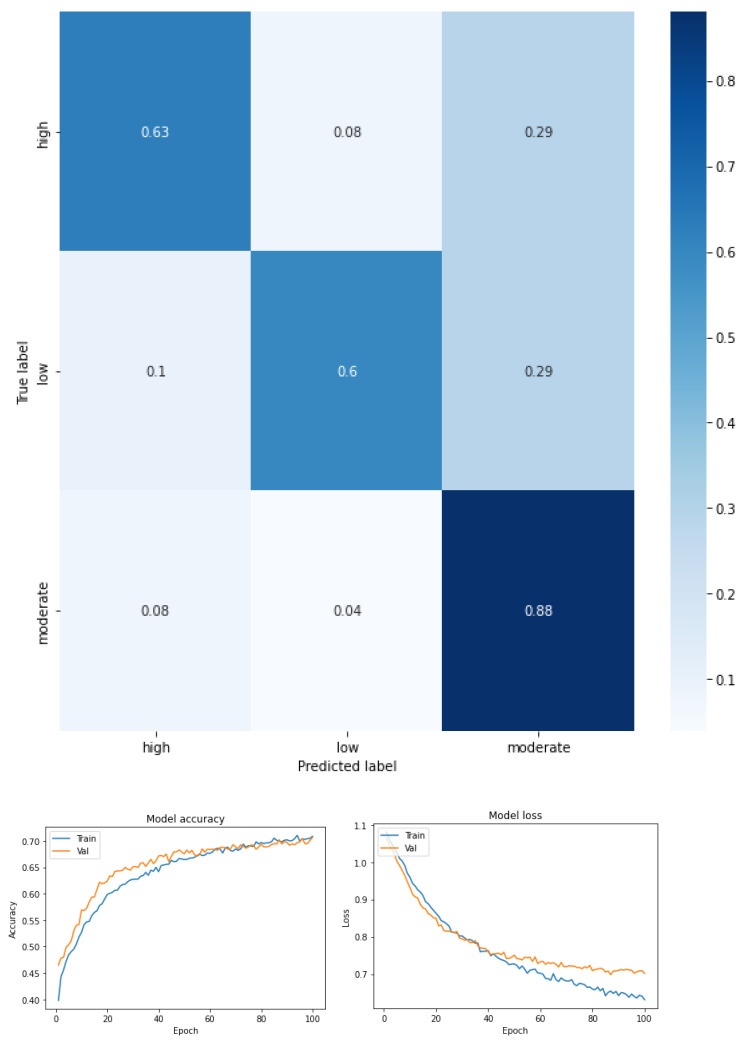
Confusion matrix, accuracy, and loss function for the total dataset (KOA mild and moderate) for the three levels of FOF.

**Figure 4:**
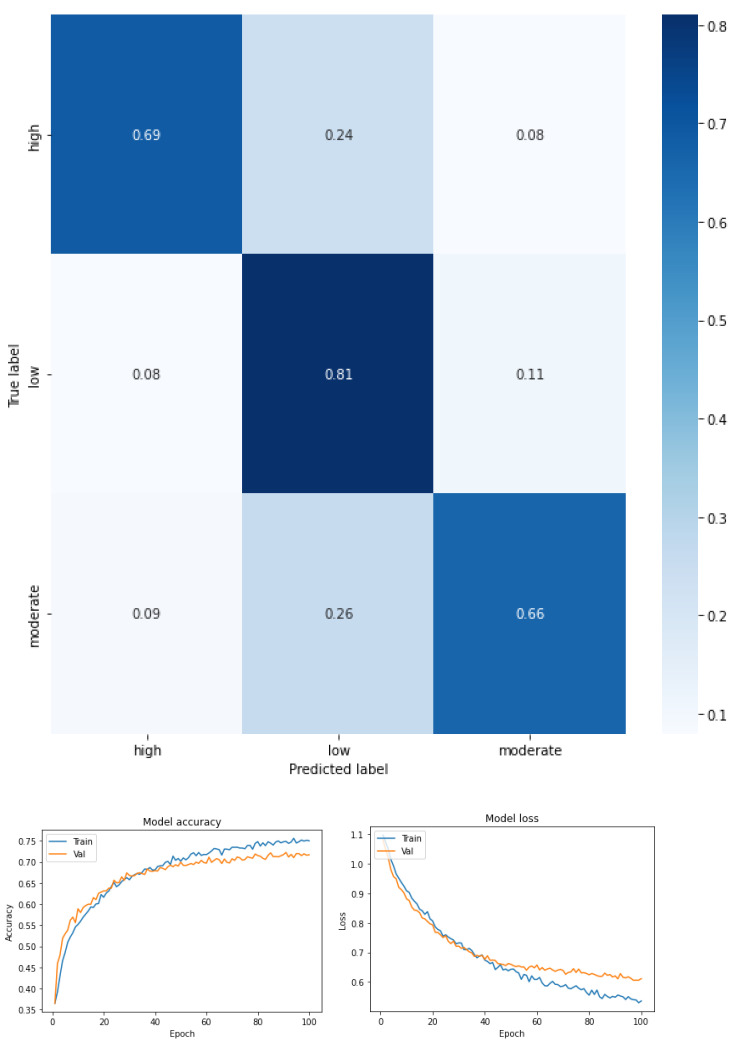
Confusion matrix, accuracy, and loss function for mild osteoarthritis and the three levels of FOF.

**Figure 5:**
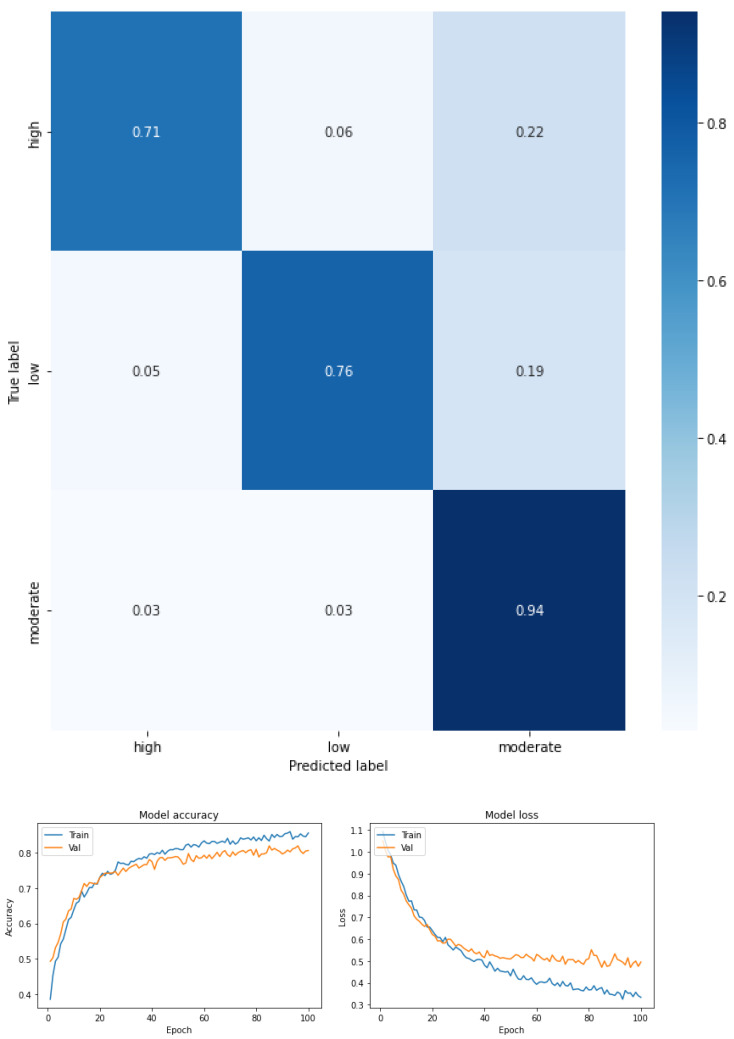
Confusion matrix, accuracy, and loss function for moderate osteoarthritis and the three levels of FOF.

**Figure 6:**
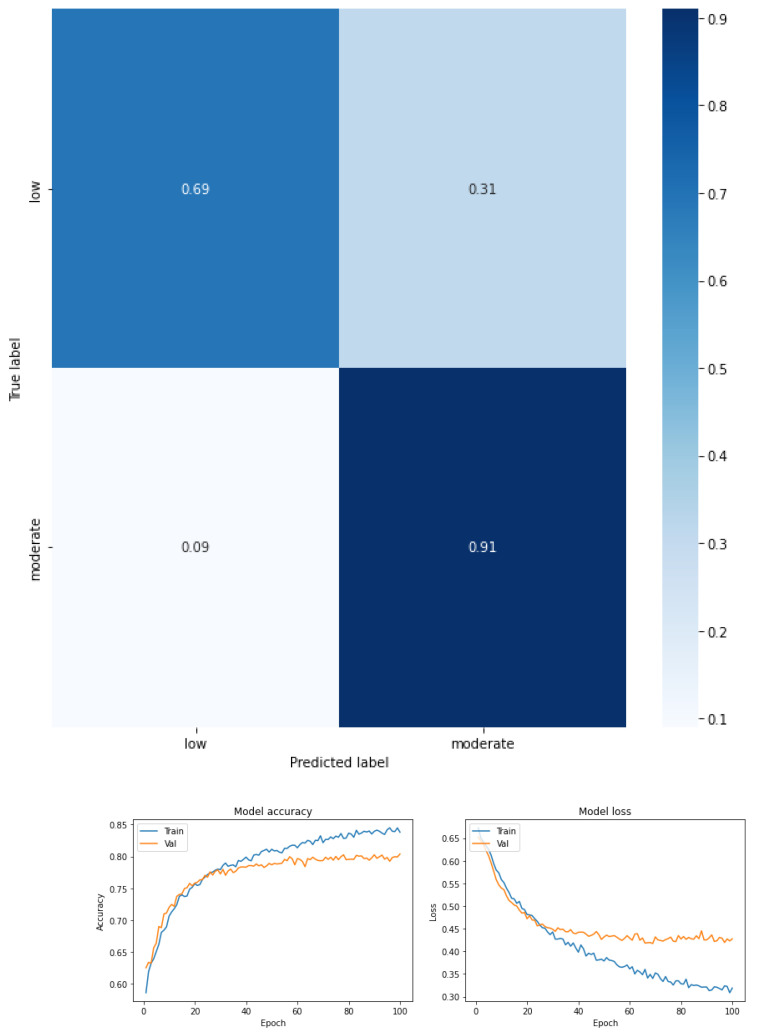
Confusion matrix, accuracy, and loss function for the total dataset for two levels of FOF (low vs. moderate).

**Figure 7:**
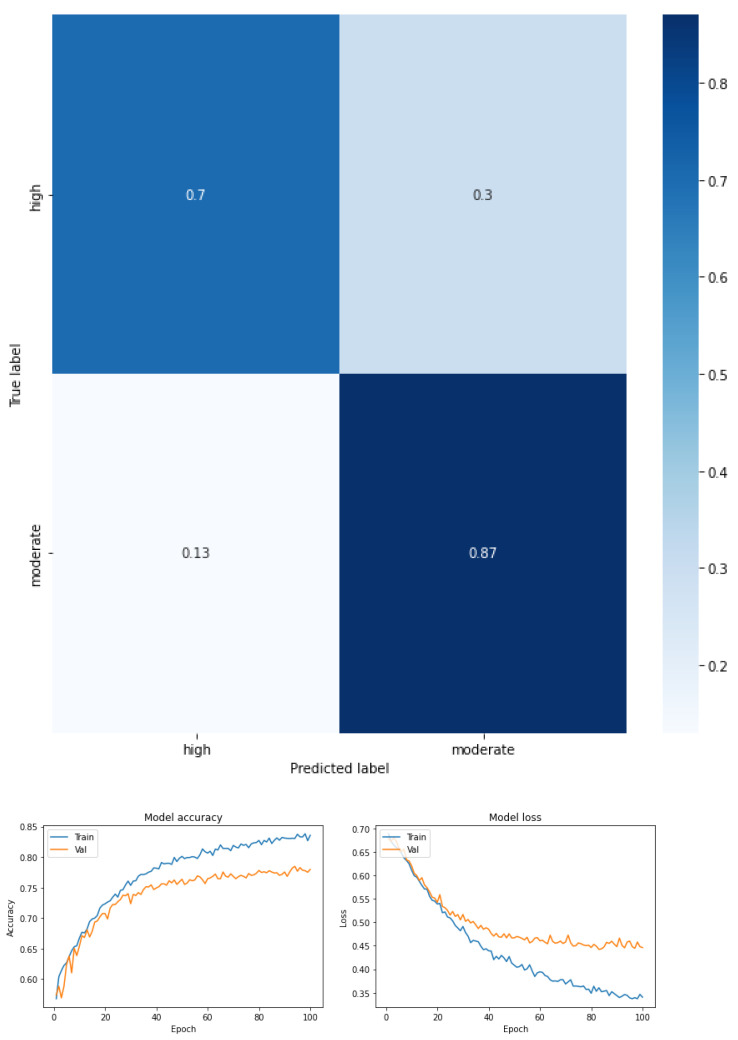
Confusion matrix, accuracy, and loss function for the total dataset for two levels of FOF (moderate vs. high).

**Figure 8:**
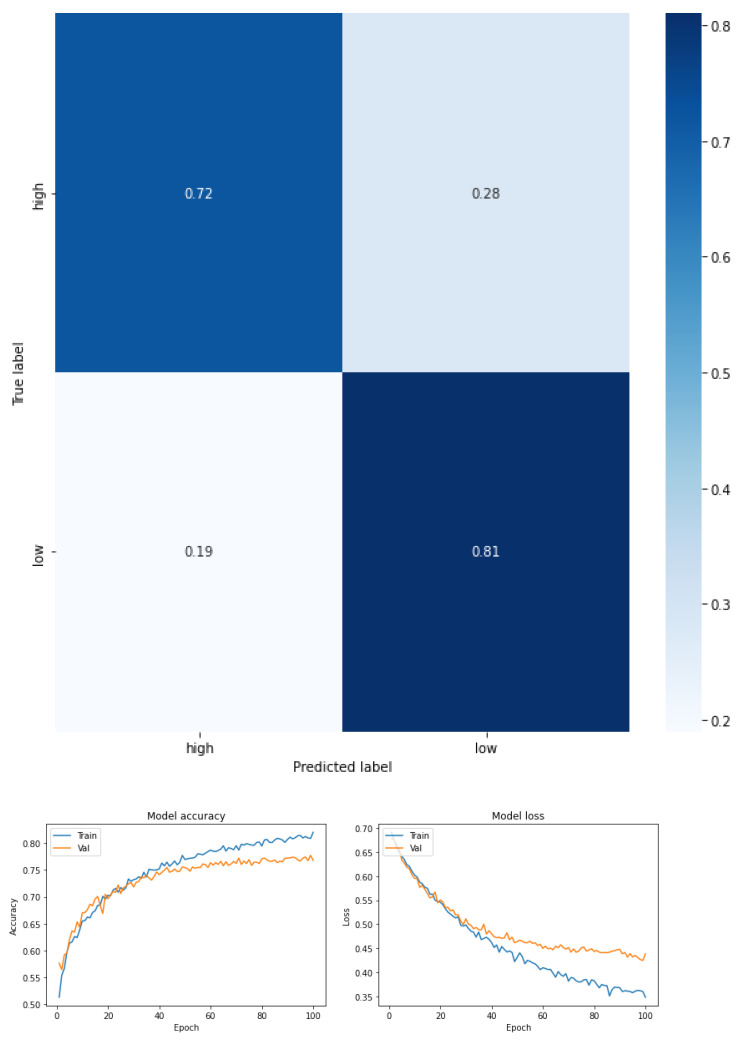
Confusion matrix, accuracy, and loss function for the total dataset for two levels of fear of fall (low vs. high).

**Figure 9:**
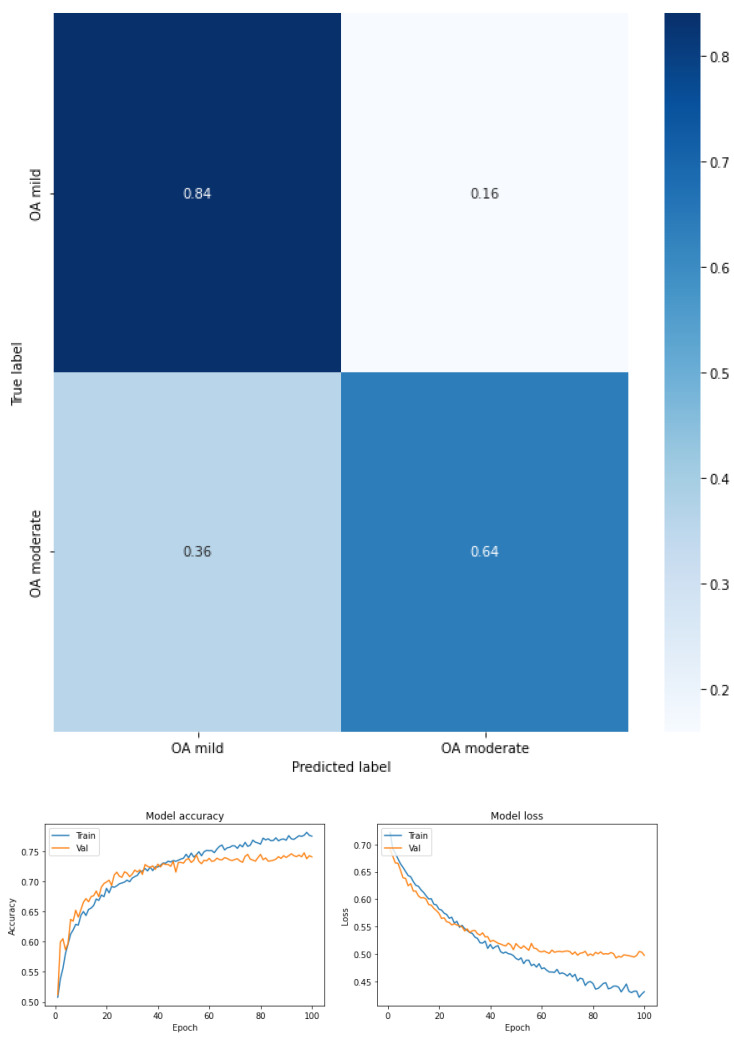
Confusion matrix, accuracy, and loss function for KOA mild vs. moderate.

- Fear of falling for three classes—low, moderate, and high—for the total dataset (KOA mild and moderate) (see Figure 3 and Table 5).
- Fear of falling for three classes—low, moderate, and high—for mild knee osteoarthritis (see Figure 4 and Table 6).
- Fear of falling for three classes—low, moderate, and high—for moderate knee osteoarthritis (see Figure 5 and Table 7).
- Fear of falling for two classes—low vs. moderate—for the total dataset (KOA mild and moderate) (see Figure 6 and Table 8).
- Fear of falling for two classes—moderate vs. high—for the total dataset (KOA mild and moderate) (see Figure 7 and Table 9).
- Fear of falling for two classes—low vs. high—for the total dataset (KOA mild and moderate) (see Figure 8 and Table 10).
- Classification between KOA mild vs. moderate (see Figure 9 and Table 11).

The results are expressed through performance metrics such as the confusion matrix and precision and loss function after 100 iterations. In addition, the precision, recovery, and F1 score values for each classification test are summarized in tables. All classes were balanced prior to classification so as not to favor any one class over another.

Figure 3 shows the confusion matrix obtained and Table 5 summarizes the values of precision, recovery, and F1 score for a multiclass classification by FOF (mild, moderate, and high) for the total study sample (considering mild and moderate KOA). As can be seen in the confusion matrix, moderate FOF is more separated from the other classes (recall 0.88), well above the values obtained in the other two classes in which there is some confusion between low and high FOF.

Precision (the model’s ability to identify the FOF classes) and recall (as the recall rate of the FOF classes that were identified) and the F1 score refers to the harmonic mean between precision and recall, i.e., a combination of both metrics to express a performance indicator with the same weight associated with precision and recall.

For this case, an F1 score of 0.70 was achieved for the high FOF class, 0.72 for moderate FOF, and 0.69 for low FOF. This means that the precision and recall values for FOF in all classes had an average score. However, the high and low classes were characterized by an imbalance favoring accuracy over recall. The moderate class, however, was characterized by the imbalance, which was reversed with higher recall relative to precision. Precision (the number of predictions that are correct out of the total number of predictions made) was 0.71. The macro-average metric was calculated as an indicator of the arithmetic mean of the classes; in this case, the values were precision 0.74, recall 0.70, and F1 score 0.71, and the weighted average metric was used as a measure of central tendency of a series of quantitative FOF observations considering the support of each class. The weighted average of the three FOF classes was precision 0.74, recall 0.71, and F1 score 0.71.

Figure 4 shows the confusion matrix obtained, and Table 6 summarizes the values of precision, recovery, and F1 score for a multiclass classification by FOF (low, moderate, and high) considering only the mild KOA condition. The confusion matrix shows that the low FOF is further separated from the other classes (recall 0.81) by almost 10 points from the values obtained in the other two classes—low FOF (recall 0.69) and high FOF (recall 0.66). On the other hand, the performance now for all the FOF values in KOA is mild. In this case, it can be seen that the model obtained a medium F1 score (0.74 high FOF, 0.70 low FOF, and 0.71 moderate FOF). For high and moderate FOF, the precision was greater than the recall, whereas for low FOF, the recall was greater than the precision. The accuracy was 0.72. The macro-average metric, the values were precision 0.73, recall 0.72, and F1 score 0.72, and the weighted average metric of the three FOF classes in KOA mild was in precision 0.74, recall 0.72, and F1 score 0.72.

Figure 5 shows the confusion matrix obtained, and Table 7 summarizes the values of precision, recovery, and F1 score for a multiclass classification by FOF (low, moderate, and high) considering only the moderate KOA condition. The confusion matrix shows a great separability of the moderate FOF class (recall 0.94) with respect to the other classes (recall 0.76 and 0.71 for low and high FOF, respectively). In this case, it can be seen that the model obtained a high F1 score (0.79 high FOF, 0.83 low FOF, and 0.80 moderate FOF). For high and low FOF, the precision was greater than the recall, whereas for moderate FOF, the recall was greater than the precision. The accuracy was 0.81. The macro-average metric, the values were precision 0.83, recall 0.81, and F1 score 0.81, and the weighted-average metric of the three FOF classes in KOA moderate was in precision 0.83, recall 0.81, and F1 score 0.81.

Figure 6, Figure 7 and Figure 8, together with Table 8, Table 9 and Table 10, show the performance metrics in which the precision, recovery, and F1 score values are summarized for a biclass classification by FOF considering the entire sample.

With regard to the classification between low and moderate FOF (see Figure 6 in this case), it can be seen that the model obtained a high F1 score (0.77 low FOF, and 0.83 moderate FOF). For low FOF, the precision was greater than the recall (0.88 and 0.69, respectively), whereas for moderate FOF the recall was greater than the precision (0.76 and 0.91, respectively). The accuracy was 0.80. For the macro-average metric, the values for precision were 0.82, recall 0.80, and F1 score 0.80. Finally, the weighted average metrics were 0.83 for precision, 0.81 recall, and 0.81 for the F1 score.

With regard to the classification between moderate and high FOF, Figure 7 shows the performance of the metrics, now for the moderate and high FOF classes in the total dataset. In this case, it can be seen that the model obtained a high F1 score (0.76 high FOF, and 0.79 moderate FOF). For high FOF, the precision was greater than the recall (0.85 and 0.70, respectively), whereas for moderate FOF the recall was greater than the precision (0.73 and 0.87, respectively). The accuracy was 0.78. For the macro-average metric, the values for precision were 0.79, recall 0.78, and F1 score 0.78. Finally, the weighted average metrics were 0.79 for precision, 0.78 recall, and 0.78 for the F1 score.

With regard to the classification between low and high FOF (Figure 8), it can be seen that the model obtained a high F1 score (0.76 high FOF, and 0.77 moderate FOF). For high FOF, the precision was greater than the recall (0.80 and 0.72 respectively), whereas for low FOF the recall was greater than the precision (0.74 and 0.81, respectively). The accuracy was 0.77. For the macro-average metric, the values for precision were 0.77, recall 0.77, and the F1 score was 0.77. Finally, the weighted average metrics were 0.77 for precision, 0.77 recall, and 0.77 for the F1 score.

Figure 9 and Table 11 show the results of the classification but by condition of KOA (mild vs. moderate) where the identification capacity of the mild KOA class is achieved with a recall of 0.84, well above the 0.64 obtained for the moderate KOA class. However, the difference in F1 score values between the two classes is minimized (0.76 for mild KOA and 0.71 for moderate KOA.

## 4. Discussion

(1) With regard to gait characteristics according to degree of FOF, accelerometric features extracted during a gait task show motor behavior in terms of magnitude of temporal–spatial variables, with a tendency to become more cautious as levels of worry or fear of falling increase. This can probably also be interpreted as a loss in the degrees of freedom of movement and lack of adaptability or variability during movement in older people. Evidence of this is that the speed in the low FOF group reached a mean = 0.752 m/s, moderate FOF 0.694 m/s, and high FOF 0.653 m/s. In other words, the speed depends on the level of FOF, specifically when it is located between low- and high-level FOF (*p* < 0.009). Speed is a relevant indicator used in the clinical field of gait recovery, being closely related to the functionality of people.

It is interesting to observe how this cautious pattern is indeed significant between the low and high levels for the speed and cadence variables. Here is a first emphasis, from the clinical point of view, in which at least the intervening variable FOF could be involved in promoting a cautious pattern and risk of falling. The Short FES-I scores (7–8: low; 9–13: moderate; 14–28: high) that classify the levels seem to respond well to comparisons between the extreme levels, but not between the moderate level. This may probably be due to the cutoff scores and psychometric characteristics of the Short FES-I.

On the other hand, performance metrics that commonly seek to characterize gait (cadence, velocity, RMS, stride length, etc.) as measures of central tendency fail to distinguish between classes in this experiment. This shows us that it is a problem of difficult separation between classes, forcing us to look for other data analysis techniques such as CNNs that demonstrate a greater capacity to address the problem by identifying patterns within the data without the intervention of the researcher.

(2) Currently, CNNs have been applied in different situations related to motor control and biological signal analysis situations that characterize human movement. In this investigation, it was important to characterize and classify center-of-mass acceleration patterns in older people with mild and moderate KOA associated with different levels of worry or FOF.

The analysis of the confusion matrices in the different comparative situations proposed allows us to adequately discriminate between the acceleration patterns of the moderate class above the low or high FOF.

The descriptive statistical analysis allows us to realize that the moderate class does not show differences in means with the extreme classes. This may be due to the psychometric nature of the short FES. However, we obtained an adequate classification by using CNNs.

By virtue of the results, we can show a good classification performance was obtained in this research among the classes of FOF, highlighting the ability to identify moderate FOF with respect to mild and high FOF independent of the KOA condition (mild, moderate, or both).

### 4.1. Limitations

The amount of data for each of the different conditions studied was not balanced by the number of subjects studied, nor by the number of values recorded in each acceleration signal. This made it necessary to balance the data according to the minority class in order to avoid over-learning of one class over another. However, this affected the amount of data studied. Another limitation of our study was the sample size. The data from a sample of 69 participants, which was a convenience, is not representative of the population. Due to the complexities of these types of designs, other investigations have also had a similar difficulty. A strategy to the above could be to explore our analysis proposal in simulated data and contribute to arguing our hypothesis.

### 4.2. Future Projections

The study of gait patterns from the clinical point of view requires quantitative methodologies that allow their better characterization and understanding. In this context, it becomes even more relevant and necessary to continue with this line of study to achieve a better characterization of the motor function of gait and its relationship with the concern about falling, which generates a functional limitation in patients. Additionally, we propose that future research may consider larger sample sizes or, alternatively, try simulated data that may contribute to an optimal generalization of the model with a greater amount of possible data.

## 5. Conclusions

Classifying center-of-mass acceleration patterns based on the clinical reference of FOF is a difficult exercise within the same pathology as KOA for any classification algorithm (machine learning or deep learning). This is mainly due to the fact that the FOF is a subjective perception and that it is not necessarily related or manifested with important changes in the motor behavior of the subject during the march, nor does it manifest itself with values very close to the border or border between label (FOF mild vs. moderate or moderate vs. high).

However, by virtue of the results, we can evidence a good classification performance obtained in this research among the classes of FOF, highlighting the ability to identify moderate FOF with respect to mild and high FOF independent of the KOA condition (mild, moderate, or both). This study explores the use of CNNs directly on the acceleration signal of the center of mass of the gait record. It offers a good alternative to machine learning models that need a significant feature extraction step for identifying gait patterns. Although the CNN model proposed in this article achieved satisfactory results, more research, using more data, is needed to study the use of such models in clinical practice. This research is innovative in this aspect, as the FOF is a challenge for both clinicians and researchers interested in understanding this phenomenon.

## Figures and Tables

**Table 1:** Sample characteristics.

FOF	KOA	KOA Moderate	Total Group (n)	Age (Years)	Weight (kg)	Size (m)	IMC	Barthel	MMSE
Low	13	4	17	74.76 ± 5.29	64.65 ± 10.78	1.50 ± 6.55	28.58 ± 5.05	98.82 ± 2.81	17.29 ± 1.86
Moderate	16	6	22	75.27 ± 5.63	67.71 ± 9.90	1.55 ± 9.07	27.91 ± 3.32	99.32 ± 1.79	17.22 ± 1.774
High	17	13	30	73.06 ± 5.34	71.23 ± 13.06	1.52 ± 8.21	30.83 ± 5.90	95.33 ± 4.9	16.83 ± 1.98
Total group (n)	46	23	69	74.18 ± 5.44	68.49 ± 11.73	143.55 ± 36.16	29.34 ± 5.10	97.46 ± 4.07	17.07 ± 1.90
*p*-value	–	–	–	0.311	0.235	0.652	0.191	0.01	0.43

**Table 2:** Short FES-I sample characteristics.

	KOA Mild			KOA Moderate		
	**Low**	**Moderate**	**High**	**Low**	**Moderate**	**High**
n	13	16	17	4	6	13
Score	7.61	11.46	17.23	7.5	10	17.92
SD	0.56	1.66	2.91	0.57	1	2.25

**Table 3:** Characteristics of the CNN model.

Layer (Type)	Parameters
conv2d_ (Conv2D)	32 filter, kernel 2 × 2, activation ReLU
dropout_6 (Dropout)	0.3
conv2d_5 (Conv2D)	64 filter, kernel 2 × 2, activation ReLU
dropout_7 (Dropout)	0.4
flatten_2 (Flatten)	
dense_4 (Dense)	128 activation ReLU
dropout_8 (Dropout)	0.5
dense_5 (Dense)	3, activation Softmax

**Table 4:** Descriptive statistics of typically reported variables of acceleration patterns on gait.

Descriptive Statistics																		
	**Velocity** **(mts/seg)**			**Cadence** **(step/min)**			**Step Length** **(cm)**			**RMS-AP**			**RMS-ML**			**RMS-VT**		
	**Low**	**Mod**	**High**	**Low**	**Mod**	**High**	**Low**	**Mod**	**High**	**Low**	**Mod**	**High**	**Low**	**Mod**	**High**	**Low**	**Mod**	**High**
	17	21	31	17	21	31	17	21	31	17	21	31	17	21	31	17	21	31
Mean	0.75	0.69	0.65	111.25	105.63	100.82	0.54	0.54	0.51	1.39	1.17	1.08	1.72	1.3	1.29	0.68	0.55	0.54
Std. Deviation	0.08	0.13	0.09	10.5	11.73	14.42	0.09	0.13	0.09	0.45	0.42	0.27	0.49	0.34	0.29	0.2	0.19	0.17
Minimum	0.60	0.41	0.44	89.9	83.6	59.14	0.41	0.37	0.37	0.84	0.36	0.6	1	0.55	0.68	0.23	0.26	0.33
Maximum	0.94	1.01	0.83	128.1	128.2	119.2	0.74	0.93	0.74	2.2	2.2	1.7	2.5	1.9	2.1	1.3	0.92	0.91

**Table 5:** Performance metrics for three classes of FOF for the total dataset.

	Precision	Recall	F1-Score
high	0.78	0.63	0.70
low	0.82	0.60	0.69
moderate	0.61	0.88	0.72
accuracy			0.71
macro avg	0.74	0.70	0.71
weighted avg	0.74	0.71	0.71

**Table 6:** Performance metrics for three classes of FOF and mild KOA.

	Precision	Recall	F1-Score
high	0.81	0.69	0.74
low	0.61	0.81	0.70
moderate	0.78	0.66	0.71
accuracy			0.72
macro avg	0.73	0.72	0.72
weighted avg	0.74	0.72	0.72

**Table 7:** Performance metrics for three classes of FOF and moderate KOA.

	Precision	Recall	F1-Score
high	0.88	0.71	0.79
low	0.91	0.76	0.83
moderate	0.69	0.94	0.80
accuracy			0.81
macro avg	0.83	0.81	0.81
weighted avg	0.83	0.81	0.81

**Table 8:** Performance metrics for two classes of FOF (low vs. moderate for the total dataset).

	Precision	Recall	F1-Score
low	0.88	0.69	0.77
moderate	0.76	0.91	0.83
accuracy			0.80
macro avg	0.82	0.80	0.80
weighted avg	0.82	0.80	0.80

**Table 9:** Performance metrics for two classes of FOF (moderate vs. high) for the total dataset.

	Precision	Recall	F1-Score
high	0.85	0.70	0.76
moderate	0.73	0.87	0.79
accuracy			0.78
macro avg	0.79	0.78	0.78
weighted avg	0.79	0.78	0.78

**Table 10:** Performance metrics for two classes of FOF (low vs. high) for the total dataset.

	Precision	Recall	F1-Score
high	0.80	0.72	0.76
low	0.74	0.81	0.77
accuracy			0.77
macro avg	0.77	0.77	0.77
weighted avg	0.77	0.77	0.77

**Table 11:** Performance metrics KOA mild vs. moderate classification.

	Precision	Recall	F1-Score
KOA mild	0.70	0.84	0.76
KOA moderate	0.80	0.64	0.71
accuracy			0.74
macro avg	0.75	0.74	0.74
weighted avg	0.75	0.74	0.74

## Data Availability

Not applicable.

## Abbreviations

The following abbreviations are used in this manuscript:

EP	Elderly people
KOA	Knee osteoarthritis
FOF	Fear of falling
